# The Endovascular Therapy for In-Stent Restenosis After Fluoropolymer-Based Drug-Eluting Stent Implantation in Femoropopliteal Lesions

**DOI:** 10.1016/j.jacasi.2024.10.023

**Published:** 2025-01-07

**Authors:** Kazunori Horie, Mitsuyoshi Takahara, Osamu Iida, Yoshimitsu Soga, Terutoshi Yamaoka, Masahiko Fujihara, Daizo Kawasaki, Shigeo Ichihashi, Norio Tada

**Affiliations:** a Department of Cardiovascular Medicine, Sendai Kousei Hospital, Miyagi, Japan; b Department of Laboratory Medicine, Osaka University Graduate School of Medicine, Osaka, Japan; c Cardiovascular Division, Osaka Police Hospital, Osaka, Japan; d Department of Cardiology, Kokura Memorial Hospital, Kitakyushu, Japan; e Department of Vascular Surgery, Matsuyama Red Cross Hospital, Matsuyama, Ehime, Japan; f Department of Cardiology, Kishiwada Tokushukai Hospital, Kishiwada, Japan; g Department of Cardiology, Morinomiya Hospital, Osaka, Japan; h Division of Radiology, Nara Medical University, Kashihara, Japan

**Keywords:** drug-coated balloon, drug-eluting stent(s), endovascular therapy, femoropopliteal disease, in-stent restenosis

The antiproliferative effects of paclitaxel and durable-polymer coating of the fluoropolymer-based drug-eluting stent (FP-DES) (Eluvia, Boston Scientific) induced an acceptable primary patency rate of 72.7% within 3 years in femoropopliteal artery lesions according to a large-scale registry.^1^ However, the 3-year incidence of in-stent occlusion and stent thrombosis were not low at 16.1% and 7.3%, respectively.^1^ No studies focused on the clinical prognosis after endovascular therapy (EVT) for in-stent restenosis (ISR) related to FP-DES. This multicenter retrospective study aimed to evaluate the 1-year prognosis after target lesion revascularization (TLR) using EVT for in-stent restenosis/occlusion related to FP-DES.

We retrospectively analyzed the database from the CAPSICUM (Contemporary outcomes After Paclitaxel-eluting peripheral stent implantation for symptomatic lower limb IsChemia with sUperficial feMoral or proximal popliteal lesion) study.^1^ The CAPSICUM study was a prospective, multicenter, observational study that registered 1,204 limbs from 1,097 patients who underwent FP-DES implantation for symptomatic atherosclerotic femoropopliteal artery disease between February 2019 and June 2020 at 67 cardiovascular centers across our country. All participants were asked to visit their centers 6, 12, 24, 36, and 48 months after FP-DES implantation to evaluate the occurrence of ISR by ultrasonography. ISR were detected in 301 limbs during a median follow-up of 23.8 months (Q1-Q3: 5.0-44.7 months). We excluded 9 limbs that underwent surgical revascularization and 73 limbs that underwent conservative management. Clinical follow-up data were not available for 19 limbs, and they were excluded in the present study. Thus, the analysis included 200 consecutive limbs from 195 patients undergoing TLR using EVT. This study was approved by the Institutional Review Board of the first author’s hospital in 2023 (approval number: 27-33). The study protocol was also approved by the local ethics committee of all participating centers. The data analysis was performed according to the Declaration of Helsinki.

The ISR lesions were classified on angiography according to the previous study, class I: focal ISR, ≤50 mm in length; class II: diffuse ISR, >50 mm in length; and class III: re-occlusion.^2^ Lesion length was measured by visual estimation, and in cases with class III lesion length was defined as the sum of ISR and in-stent occlusion length. The primary endpoint of the study was recurrent restenosis, which was defined as a peak systolic velocity ratio of over 2.4 measured with duplex ultrasound and/or >50% diameter stenosis or occlusion within the implanted stent.^1^ The secondary endpoints were the periprocedural complications and the incidences of all-cause mortality, major amputations, and clinically driven revascularization one year after EVT for ISR of FP-DES.

Technical success of EVT was defined as residual stenosis of <30% of the reference diameter without delayed antegrade blood flow, adjudicated by visual estimation. If lesions exhibited visible thrombosis, catheter-based aspiration was conducted before and/or after balloon dilatation. Target lesions were dilated using uncoated balloons at first. Drug-coated balloons (DCBs) were dilated at the discretion of each operator. Scaffolds were implanted if extensive dissection or residual stenosis was detected after balloon dilatation. The rates of freedom from events were estimated using the Kaplan-Meier analysis. The association between baseline characteristics and re-restenosis risk was analyzed using the Cox proportional hazards models with mixed effects, where the interpatient variability was treated as the random effects. We confirmed that the proportional hazards assumption was not statistically denied by the Schoenfeld residuals test (*P >* 0.05).

The mean patient age was 73 ± 10 years, and the proportion of male patients was 62.6% (122 of 195). The prevalence of end-stage renal disease requiring dialysis and chronic limb-threatening ischemia (CLTI) were 32.3% (63 of 195) and 37.0% (74 of 200), respectively. ISR of FP-DES presented as claudication (55.5%, 111 of 200), CLTI (39.0%, 78 of 200), and acute limb ischemia (ALI) (5.5%, 11 of 200). Among the claudicants (n = 126) at implantation of FP-DES, CLTI and ALI developed in 21.4% (27 of 126) and 2.4% (3 of 126) of limbs at onset of FP-DES failure. Among patients with CLTI (n = 74) at implantation of FP-DES, ALI developed in 10.8% (8 of 74) of limbs at onset of FP-DES failure. The proportions of class I, II, and III lesions were 23.2% (46 of 198), 15.7% (31 of 198), and 61.1% (121 of 198) (missing data: 1.0%). The median length of ISR was 22 cm (Q1-Q3: 12-28 cm). ISR lesions were treated using DCB (55.6%, 110 of 195), additional scaffold implantation (30.8%, 60 of 195), and plain balloon angioplasty (12.3%, 25 of 195) (missing data: 1.4%). Catheter-based thrombectomy, excimer laser, and distal protection were conducted in 40.5% (81 of 200), 8.2% (16 of 195; missing data: 2.5%), and 13.6% (27 of 198; missing data: 1.0%). Technical success of EVT for FP-DES failure was achieved in 78.8% (95% CI: 72.4%-84.3%) of lesions. Distal embolization was observed in 4.5% (95% CI: 2.1%-8.5%), and acute occlusion within 30 days after EVT occurred in 4.5% (95% CI: 2.1%-8.4%).

During a median follow-up period of 6.8 months (Q1-Q3: 0.6–17.5 months), re-restenosis was observed in 66 limbs. According to Kaplan-Meier analysis, the freedom from re-restenosis and was 65.4% (95% CI: 58.0%-73.7%) (Figure 1A). The 1-year freedom from re-TLR, limb salvage, and overall survival was 77.9% (95% CI: 71.7%-84.6%), 98.8% (95% CI: 97.2%-100.0%), and 85.4% (95% CI: 80.3%-90.7%). Figure 1B shows the association of patient and lesion characteristics with the incidence of re-restenosis. DCB use for TLR was independently associated with the prevention of re-restenosis (adjusted HR: 0.42 [95% CI: 0.24–0.74; *P =* 0.003]).

**Figure 1:**
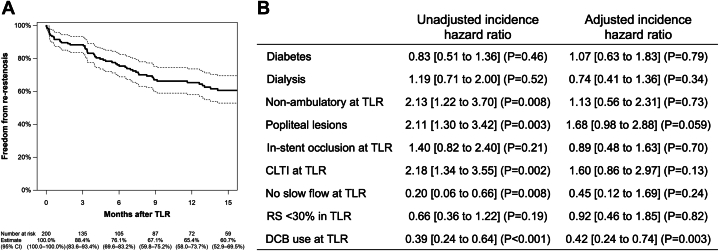
Primary Patency After TLR and Risk Factors of Re-Restenosis (A) Kaplan-Meier curve of freedom from re-restenosis. Dotted lines indicate 95% CIs. (B) The association between baseline characteristics and re-restenosis risk was analyzed using the Cox proportional hazards models with mixed effects, where the interpatient variability was treated as the random effects. The proportional hazards assumption was satisfied for the fitted model (*P >* 0.05 by the Schoenfeld residuals test). Data are HRs [95% CIs] (*P* values). CLTI = chronic limb-threatening ischemia; DCB = drug-coated balloon; RS = residual stenosis; TLR = target lesion revascularization.

The CAPSICUM registry reported that the patterns of FP-DES failure including onset of in-stent occlusion and stent thrombosis were not different between the early and late phase, suggesting that arterial repair was still incomplete and that the risk of occlusive restenosis and stent thrombosis could not be completely eliminated.^1^ A previous histopathological analysis of limb arteries in a swine model demonstrated that FP-DES use was associated with incomplete vessel healing, which included more malapposed struts with excessive fibrin deposition compared with drug-coated stents.^3^ These findings indicate that malapposed struts might provoke thrombosis formation in FP-DES,^1^ and may cause the low technical success rate of EVT and the poor primary patency. On the other hand, our study showed that the only protection from re-restenosis was use of DCB in TLR. An autopsy in a patient with FP-DES for femoropopliteal disease revealed that neointimal proliferation was noted in partial struts.^4^ In addition, Golchehr et al^5^ detected edge restenosis in 70% of patients who developed acute thrombosis after implantation of intraluminal stent grafts. Edge restenosis may increase the risk of re-occlusion also after implantation of FP-DES. Although the present study did not establish the efficacy of DCB treatment for FP-DES failure because of the retrospective study design, our results suggested that DCB might prevent recurrent restenosis in ISR related to FP-DES.

The limitation of this study was that it was a retrospective, nonrandomized, and single-arm design. The impact of lesion characteristics and EVT procedure on the patency could not be evaluated appropriately because of the small sample size. Moreover, EVT procedure was dependent on each operator’s decision without following any pre-established protocol including intravascular imaging, which might cause a bias.

We concluded that the freedom from re-restenosis was 65.4%, indicating that ISR related to FP-DES is still challenging for EVT.

## Funding Support and Author Disclosures

The CAPSICUM study was supported by the Research Association for Lower Limb Artery Revascularization (LIBERAL) sponsored by Boston Scientific Japan K.K., OrbusNeich Foundation, Terumo Corporation, and Kaneka Medix Corporation. The funding companies played no role in study design, selection of enrolled patients, treatment strategy, revascularization procedures or equipment, or collection, analysis, or interpretation of data. Drs Iida and Soga are consultants who received honoraria from Boston Scientific. Drs Yamaoka, Fujihara, Kawasaki, and Ichihashi have received remuneration from Boston Scientific Japan. All other authors have reported that they have no relationships relevant to the contents of this paper to disclose.

## References

[1] Iida O., Takahara M., Soga Y. (2024). Three-year clinical course after fluoropolymer-based drug-eluting stent implantation for femoropopliteal lesions. Vasc Med.

[2] Tosaka A., Soga Y., Iida O. (2012). Classification and clinical impact of restenosis after femoropopliteal stenting. J Am Coll Cardiol.

[3] Sakamoto A., Torii S., Jinnouchi H. (2021). Vascular response of a polymer-free paclitaxel-coated stent (Zilver PTX) versus a polymer-coated paclitaxel-eluting stent (Eluvia) in healthy swine femoropopliteal arteries. J Vasc Interv Radiol.

[4] Matsumoto Y., Torii S., Morino Y. (2022). Pathography of superficial femoral artery treated with ELUVIA^TM^ paclitaxel eluting stent. Circ J.

[5] Golchehr B., Fritschy W.M., Holewijn S. (2013). Outcome of thrombolysis and thrombectomy for thrombosed endografts inserted in the superficial femoral artery for occlusive disease. J Endovasc Ther.

